# A standardized comparison of commercially available prion decontamination reagents using the Standard Steel-Binding Assay

**DOI:** 10.1099/vir.0.027201-0

**Published:** 2011-03

**Authors:** Julie Ann Edgeworth, Anita Sicilia, Jackie Linehan, Sebastian Brandner, Graham S. Jackson, John Collinge

**Affiliations:** MRC Prion Unit, Department of Neurodegenerative Disease, UCL Institute of Neurology, Queen Square, London WC1N 3BG, UK

## Abstract

Prions are comprised principally of aggregates of a misfolded host protein and cause fatal transmissible neurodegenerative disorders of mammals, such as variant Creutzfeldt–Jakob disease in humans and bovine spongiform encephalopathy in cattle. Prions pose significant public health concerns through contamination of blood products and surgical instruments, and can resist conventional hospital sterilization methods. Prion infectivity binds avidly to surgical steel and can efficiently transfer infectivity to a suitable host, and much research has been performed to achieve effective prion decontamination of metal surfaces. Here, we exploit the highly sensitive Standard Steel-Binding Assay (SSBA) to perform a direct comparison of a variety of commercially available decontamination reagents marketed for the removal of prions, alongside conventional sterilization methods. We demonstrate that the efficacy of marketed prion decontamination reagents is highly variable and that the SSBA is able to rapidly evaluate current and future decontamination reagents.

## INTRODUCTION

Transmissible spongiform encephalopathies or prion diseases are a closely related group of fatal neurodegenerative disorders that affect the central nervous system (CNS) of mammals. They include Creutzfeldt–Jakob disease (CJD), Gerstmann–Sträussler–Scheinker disease, fatal familial insomnia and kuru in humans, bovine spongiform encephalopathy (BSE) in cattle and scrapie in sheep.

According to the protein-only hypothesis (Griffith, 1967) the infectious agent, or prion, is composed of aggregated forms of a non-native conformer of host-encoded cellular prion protein (PrP^C^), known as PrP^Sc^ (Prusiner, 1982; Collinge, 2001). PrP^Sc^ is deposited in brain and lymphoreticular tissue as stable aggregates. Prions can be generated sporadically, as a result of an as yet uncharacterized stochastic event causing PrP^C^ to PrP^Sc^ conversion, or by dominant mutations in the gene encoding PrP (*PRNP* in humans), producing mutant PrP^C^ that is hypothesized to more readily undergo spontaneous conversion to PrP^Sc^. However, uniquely among neurodegenerative disorders, prion disease can also be caused through infection with exogenous prions; the latter inducing host-encoded PrP^C^ to undergo conformational change, via seeding or template-directed refolding and hence replication and spread (reviewed by Collinge & Clarke, 2007).

Classical (sporadic) CJD is rare with the infectious material being largely confined to the tissues of the CNS (Wadsworth *et al.*, 2001; Glatzel *et al.*, 2003; Head *et al.*, 2004). Hence, the risk of iatrogenic transmission from surgical instruments has previously been considered to be relatively low. However, recent epidemiological studies have suggested an association between surgical treatment and sporadic CJD (Collins *et al.*, 1999; Mahillo-Fernandez *et al.*, 2008; de Pedro-Cuesta *et al.*, 2010). In contrast, in variant CJD (vCJD), PrP^Sc^ and infectivity have been shown to be present in a wide variety of tissues throughout the body in addition to the CNS, these include the lymphoreticular system (spleen, tonsils and lymph nodes), components of the eye and optic nerve, and in the gastrointestinal tract (Wadsworth *et al.*, 2001; Bruce *et al.*, 2001; Head *et al.*, 2004; Joiner *et al.*, 2005; Wadsworth *et al.*, 2007; Notari *et al.*, 2010). Recent cases of transfusion-related vCJD indicate that blood contains significant levels of infectivity and suggests transfusion may be an efficient route of vCJD transmission (Llewelyn *et al.*, 2004; Peden *et al.*, 2004; Wroe *et al.*, 2006; HPA Press Office, 2009). Treatment with blood products is also a risk factor and a case of vCJD prion infection has recently been reported in a haemophiliac (Peden *et al.*, 2010). With such widespread distribution of infectivity throughout the body many common surgical procedures, such as abdominal surgery, tonsillectomy, appendicectomy, gastrointestinal endoscopy and biopsy may also pose a risk for iatrogenic transmission (Collins *et al.*, 1999; Hill *et al.*, 1999; Wadsworth *et al.*, 2001; Bruce *et al.*, 2001; Bramble & Ironside, 2002; Joiner *et al.*, 2002; Wadsworth *et al.*, 2007).

Accurate risk assessment is hampered by a lack of accurate prevalence data for vCJD prion infection. A national prevalence study was carried out to estimate the number of people in the UK who are infected. Appendix material was tested for the deposition of disease-associated PrP by immunohistochemistry (Hilton *et al.*, 2004). Three positive samples were identified in a sample set of 12 674 and the authors estimated (95 % confidence interval) between 49 and 692 people per million of the population may be asymptomatic vCJD prion-infected carriers in the UK. An extensive study of discarded tonsil specimens in the UK, following on from an earlier pilot study (Frosh *et al.*, 2004), although negative, has not been able to narrow these estimates by a major degree and further studies are in progress (Clewley *et al.*, 2009).

Prion infectivity has been shown to bind avidly to steel surfaces and can effectively transmit disease to experimental animals following even brief exposure to prion-infected tissues (Zobeley *et al.*, 1999; Flechsig *et al.*, 2001). Accidental transmission to humans via a contaminated neurosurgical instrument has also been reported (Bernoulli *et al.*, 1977; Gibbs *et al.*, 1994). Collectively, all prion diseases raise the potential for surgical instruments to become contaminated with prions during surgical procedures (Davanipour *et al.*, 1985; Collins *et al.*, 1999; Minatogawa & Kumoi, 1999; Ward *et al.*, 2002; Mahillo-Fernandez *et al.*, 2008), including anaesthesia (Hernandez-Palazon *et al.*, 1998) and dentistry (Smith *et al.*, 2002; Walker *et al.*, 2007).

It has long been established that prions are resistant to conventional autoclaving as well as other sterilization techniques such as exposure to both ionizing and UV radiation and formalin treatment, which have been used in hospital environments for non-autoclavable surgical instruments (Taylor, 1991; Rutala & Weber, 2001; Fichet *et al.*, 2004). Effective destruction can be achieved by the use of corrosive agents such as 2 M NaOH or 20 % (v/v) sodium hypochlorite (Prusiner *et al.*, 1981; Taylor *et al.*, 1999). More recently, use of enzyme-based decontamination methods have shown to be equally effective (Fichet *et al.*, 2004; Jackson *et al.*, 2005; Edgeworth *et al.*, 2009) with the advantage of being applicable to delicate surgical instruments and medical devices that cannot be autoclaved, as well as being far less hazardous to operators.

An independent study examining the efficacy of routine decontamination of surgical instruments from hospitals highlighted the high levels of residual protein remaining on supposedly clean instruments (Murdoch *et al.*, 2006). Several studies have investigated the efficacy of combining detergent preparations to aid general instrument cleaning with and without the addition of enzymes as decontamination reagents for prions from surgical steel wires (Yan *et al.*, 2004; Fichet *et al.*, 2004; Jackson *et al.*, 2005; Peretz *et al.*, 2006). This research led to the launch of products designed to reduce the risks of iatrogenic prion transmission.

Here, we report on a comparative analysis of some of these commercially available prion decontamination methods using the *in vitro* Standard Steel-Binding Assay (SSBA) (Edgeworth *et al.*, 2009), which is an adaptation of the Scrapie Cell Assay (Klohn *et al.*, 2003) and is capable of sensitively detecting metal-bound prion infectivity. The assay has a large dynamic range (of ∼6 logs) and is approximately 100-fold more sensitive than conventional rodent bioassay. It is capable of detecting infectivity in brain homogenate diluted by up to 10^10^-fold (Edgeworth *et al.*, 2009), thus allowing a direct comparison of the efficacy of these reagents in decontaminating prion-infected surgical steel surfaces.

## RESULTS

### Comparison of proteinase K (PK) digestion kinetics of vCJD and RML (Rocky Mountain Laboratory) prions

**Fig. 1.**
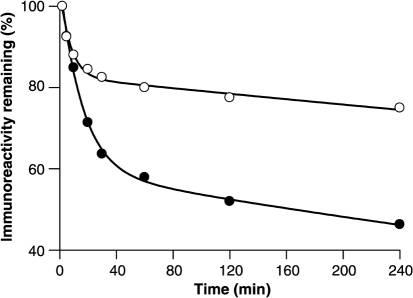
PK digestion kinetics of RML- (open circles) and vCJD-infected brain homogenates (closed circles). Following digestion with PK for varying times, samples of RML- or vCJD-infected brain homogenate were subjected to Western blotting and the levels of PrP immunoreactivity were quantified by densitometry. The values were then plotted as a percentage of the starting material with respect to time. Superimposed upon each dataset is a line representing a fit of the data to a double exponential decay.

While validation of decontamination protocols using vCJD prions would be preferable in principle, significant transmission barriers preclude sensitive bioassay in rodents, including humanized transgenic mice (Hill *et al.*, 1997; Asante *et al.*, 2002). No cell line in which vCJD prions can be efficiently propagated and assayed has yet been reported. The N2aPK1 cell line used in the SSBA that forms the basis of this study is highly susceptible to infection with RML prions, one of the best characterized and most widely used rodent-adapted prion strains (Klohn *et al.*, 2003). While RML prions seem highly suitable as a model strain on this basis, it is important when considering efficacy of decontamination reagents involving proteolysis to ensure that the disease-associated PrP is not unusually protease sensitive when compared to vCJD prions. We measured the kinetics of proteolysis of both RML and vCJD prion-infected brain homogenates by PK digestion (Fig. 1a). It was observed that disease-associated PrP from both RML and vCJD prion-infected brain homogenates were degraded at two distinct rates by PK, indicating the presence of at least two distinct subpopulations of protease-resistant material. The slowly degraded, most PK-resistant, fraction constituted more than 80 % of the material in an RML prion-infected brain, whereas this fraction was the minority of material in human vCJD prion-infected brain, where it comprised less than 47 % of the total. Additionally, the more resistant fraction associated with the RML strain type was degraded at a slower rate than the equivalent fraction associated with vCJD (0.0004 s^−1^ compared with 0.0011 s^−1^), indicating a greater resistance to degradation by PK (Table 1). While these data do not directly determine the relative sensitivities of RML and vCJD prions to the formulations tested, it does provide reassurance that RML PrP^Sc^ is not unusually protease sensitive in comparison to that seen in vCJD. We therefore considered RML prions to be a suitable model strain to assess decontamination with proteolytic methods.

### Direct comparison of commercially available decontamination reagents

**Fig. 2.**
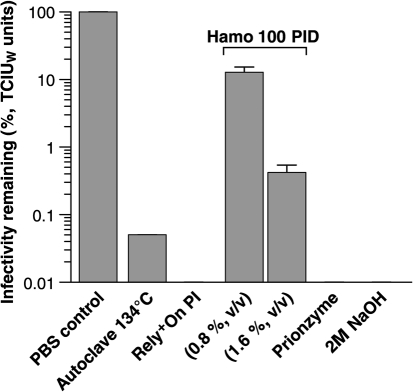
Comparison of commercial decontamination reagents by SSBA. Data are shown as the percentage of apparent TCIU_W_ units remaining on wire after treatment. The ‘% infectivity remaining’ is calculated as a percentage of the titre of infectivity assayable on wires exposed to 10^−2^ dilution of RML prion-infected brain homogenate after immersion in PBS for 1 h at room temperature. Data are presented as mean±sem. The sem for autoclaving is zero. Rely^+^On PI, Prionzyme, 2 M NaOH (the carrier for Prionzyme) decontaminate steel wires to a level beyond detection by the SSBA and therefore a reduction of prion infectivity of at least 8 logs is achieved.

These reagents were compared directly in the SSBA to quantify any remaining infectivity bound to the metal surface after treatment (Fig. 2). The most effective commercially available decontamination reagents of those tested were Rely^+^On PI (Du Pont Corporation) and Prionzyme (Genencor). Wires which were initially exposed to a 10^2^-fold dilution of RML prion-infected brain that were subsequently treated with both reagents had their residual infectivity reduced to below the detection limit of 0.0029 tissue culture infectious units on wires (TCIU_w_) (equivalent to 10^−10^ dilution of RML-infected brain). Both reagents decontaminate steel wires to a level beyond detection by the SSBA and therefore a reduction of prion infectivity of at least 8 logs is achieved. However, the decontaminating effect of Prionzyme (Genencor) is indistinguishable from that of the diluent in which Prionzyme (Genencor) is prepared (2 M NaOH solution; as per manufacturer's instructions) as treatment with 2 M NaOH alone resulted in no detectable infectivity remaining on the steel surface (Table 2).

The next most efficient method tested was autoclaving at 134 °C for 18 min in steam permeable autoclave bags [as stated in the WHO guidelines for prevention of iatrogenic transmission of vCJD (WHO, 1999)], following which we detected 0.03 TCIU_w_ units bound to the steel wire implying 95 % of detectable surface-bound prion infectivity is destroyed (Table 2). Although effective in this instance, autoclaving has previously been shown to produce highly variable results depending upon the accessibility of surfaces to steam (Jackson *et al.*, 2005). Previous studies have also demonstrated that such a titre of remaining infectivity is still sufficient to transmit prion disease to laboratory animals (Taylor *et al.*, 1998; Jackson *et al.*, 2005).

The least effective decontamination reagent of those tested was HAMO 100 PID (Steris). The HAMO 100 PID reagent was prepared at 0.8 % (v/v) and 1.6 % (v/v) (as recommended by the manufacturer) and the remaining infectivity on the steel surface determined as being equivalent to 13 and 0.4 %, respectively, of the maximum measurable bound infectivity, which equates to 0.3 and 0.07 TCIU_w_ units, respectively. Infected wires which were submerged into PBS for 1 h at room temperature as a control displayed no detectable reduction in the titre of prion infectivity bound (Table 2).

Although exposure of metal-bound prions to very high temperatures is capable of reducing the titre substantially, and treatment of such surfaces with 2 M NaOH or 20 % (v/v) sodium hypochlorite has been shown to eliminate detectable infectivity, these are not methods suitable for decontaminating delicate and valuable surgical or medical instruments that incorporate soft metals and plastics and 2 M NaOH is also a highly hazardous solution to facility operators in a central sterile services department.

### End-point titration of RML infectivity bound to steel surfaces *in vivo*

To determine the sensitivity of the steel wire bioassay for RML prions *in vivo* in tga20 mice, and hence the efficacy of the decontamination procedures, an end-point titration was performed. This allowed us to estimate that the dilution of RML prion-infected brain leading to 1 LD_50_ wire unit bound is ∼10^−5.5^. Wires exposed to a dilution of 1×10^−6^ of RML prion-infected brain homogenate are therefore estimated to have the equivalent to 0.3 LD_50_ wire units bound. Based on this calculation it can therefore be extrapolated that a wire exposed to 10^−1^ dilution of RML-infected brain can maximally harbour a load of 10^5.5^ LD_50_ intracerebral units per wire. These data are in close agreement with the findings of Lemmer *et al.* (2008), who titred the hamster-adapted scrapie strain Sc237 on steel wires implanted intracerebrally (i.c.) into hamsters. These combined data suggest that the limit of detection of prions bound to steel wires via intracerebral implantation in rodents is 0.3 LD_50_ units per wire and is likely to be a function of the wire surface area.

### *In vivo* analysis of steel wire decontamination by Rely^+^On PI

We then proceeded to further investigate these reagents by using mouse bioassay of prion-infected wires subjected to decontamination. As we have previously studied the effect of autoclaving, have also demonstrated 2 M NaOH to be effective in mouse bioassay (Jackson *et al.*, 2005), and since HAMO 100 did not sterilize the wires as assessed by SSBA, we did not consider it appropriate to study these methods further in laboratory animals.

Batches of steel wires were exposed to a 10^−1^ dilution of RML prion-infected brain [I6200, 10 % (w/v) brain homogenate containing 10^8.3^ LD_50_ units ml^−1^] and treated with Rely^+^On PI. Wires were then implanted i.c. into tga20 mice and observed for up to 250 days post-implantation. No animals were observed to develop any clinical signs of prion disease and analysis of brain tissue from all these animals by histology, PrP immunohistochemistry and Western blotting failed to detect any pathology or abnormal PrP deposition, thereby excluding detectable subclinical infection. It is therefore possible to estimate that Rely^+^On PI reduced the titre of wire bound infectivity by 5.5 logs, or to less than 0.5 LD_50_ units, since if any infectivity remained it was below the detection limit of the bioassay (Table 3). These data support the findings of the SSBA and exclude the possibility that the decontaminating reagent merely altered the strain properties of RML prions, rendering them undetectable by the N2aPK1 cells (Klohn *et al.*, 2003) used in the cell-based SSBA.

## DISCUSSION

It is well documented that prions bind avidly to many surfaces, in particular stainless-steel (Zobeley *et al.*, 1999; Flechsig *et al.*, 2001). This fact, coupled to the unusually high resistance of prions to standard decontamination methods, the widespread tissue distribution of infectivity (Wadsworth *et al.*, 2001; Bruce *et al.*, 2001; Wadsworth *et al.*, 2007) and uncertain population prevalence of vCJD prion infection, leaves ongoing public health concerns about iatrogenic transmission of vCJD prion infection in the hospital setting. Recent epidemiological evidence suggesting a proportion of apparently ‘sporadic’ or classical CJD may be linked to general surgery is exacerbating these concerns (Collins *et al.*, 1999; Mahillo-Fernandez *et al.*, 2008; de Pedro-Cuesta *et al.*, 2010). In response to such concerns, much publically funded research was performed in the UK and elsewhere and a variety of companies have invested in the development of prion decontamination reagents. Here, we have exploited the highly sensitive SSBA (Edgeworth *et al.*, 2009), developed for the quantitative analysis of prions bound to steel surfaces, to compare commercially available decontamination reagents directly.

By end-point titration of RML prion-infected brain homogenate on steel wires i.c. implanted into tga20 indicator mice, we have estimated the maximal loading capacity of a 5 mm (USP 4/0) steel wire as being 10^5.5^ LD_50_ units (Table 3), which limits the sensitivity of bioassay using rodents. Our findings are in close agreement with the results of Lemmer *et al.* (2008), who used a distinct prion strain, hamster scrapie-adapted Sc237, on i.c. implanted wires and also concluded the maximal loading capacity of wires i.c. implanted to be 10^5.5^ LD_50_ units. This therefore suggests the detection limit for prion infectivity presented on steel wires may be independent of prion strain to which the wires have been exposed.

The SSBA used here for the comparison of commercially available prion decontamination reagents is capable of detecting infectivity, resulting from exposure of steel wires to a sample containing 0.025 LD_50_ units ml^−1^ of RML compared with mouse bioassay where the limit of detection is ∼2500 LD_50_ units ml^−1^ (Table 2). The SSBA allows assessment of decontamination over a ∼8 log range.

The WHO recommended protocols for the control for iatrogenic transmission of prions that include: immersion in freshly prepared 1 M NaOH, or NaOCl, at a concentration exceeding 20 000 p.p.m. available chlorine, for 1 h at 20 °C, or porous load autoclaving at 134 °C for 18 min (WHO, 1999). However, autoclaving is not always an effective method for the decontamination of prion-infected surgical steel instruments (Taylor *et al.*, 1998; Jackson *et al.*, 2005) due to the variable ingress of superheated steam and the use of 2 M NaOH or NaOCl are not viable options for devices containing soft metals, rubbers and plastics, and are hazardous to staff. Here, we demonstrate that steel contaminated with RML prions can still harbour 5 % of contaminating infectivity after autoclaving at 134 °C for 18 min. This level of bound infectivity is still sufficient to transmit prion disease when implanted into the brains of indicator mice (Jackson *et al.*, 2005) and may still pose a risk of iatrogenic transmission in a hospital setting.

The least effective reagent for prion decontamination tested in this study was the alkali/detergent reagent HAMO 100 PID. This commercial product when tested at both the recommended (0.8 % v/v) and twice the recommended concentration (1.6 % v/v) failed to thoroughly decontaminate the steel wire surfaces, leaving 13 and 0.4 % of maximally detectable infectivity on the wires.

The most effective of the prion decontamination reagents tested were the proteolytic reagents Rely^+^On PI (DuPont Corporation) and Prionzyme (Genencor). Both reagents decontaminated the wires contaminated with RML prions to beyond the detection limit of the SSBA. The manufacturers of Prionzyme (Genencor) recommend preparation of the reagent in 2 M NaOH alkali carrier, which alone reduced the titre of infectivity bound to steel surfaces beyond the detection limit of the SSBA. This made it impossible to determine if the Prionzyme had an effect in addition to that of the 2 M NaOH in which it had been prepared (Table 2). The preparation of Prionzyme in 2 M NaOH also calls into question how practical its use would be.

A persistent problem in establishing the relative efficacy of methods for prion decontamination has been the absence of a sensitive and relevant assay that can be used as a standard for comparison. This study has evaluated several novel commercially available reagents as well as established methods of decontamination under comparable conditions in an extremely sensitive cell culture-based assay. Each of these decontamination reagents are claimed by their manufacturers to reduce prion infectivity by several logs to beyond the detection method used. However, the validation of each of these reagents has been performed using different methodologies, making direct comparison of published results difficult. The different methods have encompassed various prion strains [all of which have different proteolytic and thermodynamic properties (Somerville *et al.*, 2002)], assays with variable detection limits, and different starting materials used to provide the prion infectivity (macerates, homogenates or steel bound). While it has been suggested that the mouse-adapted BSE strain 301V may be a more suitable model to study vCJD prion inactivation (SEAC, 2006), passage of a prion strain in a new species may unpredictably affect its physicochemical properties and indeed this has recently been shown to be the case with 301V, which differs markedly in its inactivation properties to cattle BSE (Giles *et al.*, 2008). In order to assess and validate enzymic approaches to prion decontamination the selection of a suitable prion strain is crucial. The use of RML-infected material as a model substrate offers the advantage of containing material with greater resistance to proteolysis than vCJD material, which is also present in greater proportion.

This study provides a standardized method by which to compare the efficacy of reagents and methods for the decontamination of surgical instruments and offers an assay with a high dynamic range and sensitivity, beyond that of conventional rodent bioassay.

## METHODS

### Kinetic analysis of PK digestion of RML and vCJD brain homogenates.

RML and vCJD prion-infected brain homogenates (10 %, w/v) were clarified by centrifugation at 80 ***g*** for 1 min. Supernatants were removed and pre-warmed to 37 °C for 10 min. Aliquots (20 μl) were removed and mixed with an equal volume of 2× SDS loading buffer [Tris-HCL, 20 % (v/v) glycerol pH 6.8 containing 4 % (w/v) SDS, 4 % (v/v) 2-mercaptoethanol, 8 mM 4-(2-aminoethyl)-benzonase sulfonyl fluoride and 0.02 % (w/v) bromophenol blue] and snap frozen in liquid nitrogen, time point 0. PK (BDH) was added to remaining homogenates to a final concentration of 50 μg ml^−1^. Samples were incubated with agitation at 37 °C from which 20 μl aliquots were removed after 2, 5, 10, 15, 20, 30, 40, 60, 80, 120, 160, 200 and 240 min and treated as before. Samples were analysed by Western blotting as described previously (Wadsworth *et al.*, 2001) using primary antibody ICSM 35 at 0.2 μg ml^−1^ in PBS/1 % Tween-20 (PBST). Total PK-resistant PrP was determined using densitometry.

### Culturing and storage of N2aPK1 cells.

The N2aPK1 cell-line that is highly susceptible to infection with the RML prion strain (Klohn *et al.*, 2003) was used throughout the study. Cells were cultured in Opti-MEM-10 % FCS and 1 % penicillin/streptomycin (OFCS) (Invitrogen).

Individual vials containing 3 million cells of expanded subclones of the N2aPK1 cells were thawed from stocks kept in liquid nitrogen in OFCS and 6 % (v/v) DMSO and cultured for 10 days prior to incubation with RML-coated wires. Cells were cultured for no more than four passages prior to use in the SSBA (Edgeworth *et al.*, 2009) as prolonged subculturing leads to reduced susceptibility to infection.

### Preparation of homogenates.

Non-infected mouse brain homogenates were prepared from *Prnp^0/0^* mice (Bueler *et al.*, 1993), which had been back-crossed onto an FVB/NHsd background (Asante *et al.*, 2009) as 20 % (w/v) in PBS by passing through 21G to 26.5 G needles successively. Homogenate was stored as 20 μl aliquots at −80 °C. RML-infected brain homogenate (designated inocula number I6200) was prepared and titred as described previously (Cronier *et al.*, 2008). I6200 has a titre of 10^8.3^ LD_50_ units ml^−1^ of 10 % (w/v) brain homogenate.

### Preparation of steel wires for cell-based assay.

Steelex monofilament wires, USP 4/0 were cut to 2.5 cm lengths for cell-culture or 5 mm lengths for bioassay. Batches of 100 wires were placed into 50 ml falcon tubes containing 20 ml 2 % (v/v) Triton X-100 in deionized water. Wires were washed on a rocking platform for 2 h at room temperature and then washed for 5×15 min in 50 ml deionized water. Wires were then sterilized in 70 % (v/v) ethanol in deionized water for 10 min and air-dried in a class 2 microbiological safety cabinet.

### Coating of steel wires and SSBA.

Wires were placed into 1 ml Eppendorf tubes containing 1 ml inocula I6200 [10^8.3^ LD_50_ units ml^−1^ 10 % (w/v) brain homogenate] serially diluted into 10^−4^ *Prnp^0/0^* brain homogenate prepared in Opti-MEM/10 % (v/v) FCS. Steelex monofilament wire segments (60 segments of 2.5 cm) were gently agitated for 30 s and then incubated with the required RML brain homogenate dilution for 2 h at room temperature. Wires were washed in PBS containing 1 % penicillin/streptomycin for 5×15 min on a rotary wheel, air-dried and placed at a maximum of 20 wires per well into separate wells of a six-well tissue culture plate (Corning) and covered with 300 000 N2aPK1 cells. SSBA was performed as described previously (Edgeworth *et al.*, 2009).

### Decontamination of wires.

To create a reference curve, wires were exposed to a logarithmic dilution series (10^−3^–10^−9^) of I6200 in 10^−4^ FVB/N-*Prnp^0/0^* brain homogenate for 2 h and assayed by the SSBA as described previously (Edgeworth *et al.*, 2009). Batches of wires for decontamination were exposed to 1 ml 10^−1^ dilution of I6200 prepared as above. Wires were immersed into decontamination solutions, which were freshly prepared and used as directed by the manufacturer.

Prionzyme (Genencor) was prepared as 2 % (v/v) in 2 M NaOH (pH 12) and wires were incubated for 30 min at 60 °C.

HAMO 100 Prion Inactivating Detergent (HPID; Steris) was prepared at either 0.8 % (v/v) (recommended concentration by manufacturer) or 1.6 % (v/v) in deionized water and wires were incubated at 43 °C for 15 min.

Rely^+^On PI (DuPont Corporation) was prepared as a 5× stock by mixing 4.05 g pack A+2.5 g pack B+0.4 g pack C in a total volume of 50 ml of 50 °C deionized water for 10 min. Stock was then diluted to 1× working solution in deionized water in which wires were incubated in decontamination solution for 10 min at 50 °C.

For comparison, infected wires were decontaminated by autoclaving at 134 °C for 18 min in steam permeable bags or submerged into 2 M NaOH for 30 min at 20 °C. As a negative control, infected wires were immersed into PBS for 1 h at room temperature.

Wires were then rinsed in PBS at room temperature and dried before exposure to N2aPK1 cells. Cells were harvested from wires after a 3 day exposure, seeded at 4000 cells per well and assayed by SSBA (Edgeworth *et al.*, 2009), using 16 replica wells per sample.

### Mouse bioassay of RML prion-infected wires.

Steelex monofilament wire segments (60 segments of 5 mm) were prepared as described above for the SSBA. Wires were inserted i.c. into groups of tga20 mice (Fischer *et al.*, 1996) as described previously (Flechsig *et al.*, 2001).

Wires used for testing the decontamination reagent Rely^+^On PI (Du Pont Corporation), unlike those used for the dilution series, were not rinsed in PBS prior to exposure to the decontamination reagent so as to provide a more stringent test of decontamination. Wires were exposed to Rely^+^On PI for 10 min at 50 °C according to the manufacturer's instructions. Briefly, rinsed in PBS to remove residual decontamination reagent, air-dried, and then inserted i.c. into a group of 20 tga20 mice.

### Estimation of LD_50_ units on wires.

This was as described previously (Edgeworth *et al.*, 2009); briefly, the mean number of tissue culture infectious units (TCIU) per well, m, is calculated using the Poisson distribution according to the equation:
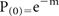
where P_(0)_ is the number of non-infected wells/total number of wells.

The value of m as determined by the wire (w) assay under standard assay conditions (TCIU_w_ per well) can be translated into apparent LD_50_ units on wire (where one LD_50_ unit represents the dose that would be lethal to 50 % of animals inoculated by the intracerebral route) by exposing wires to serial dilutions of a brain homogenate that has previously been titrated by intracerebral injection into wild-type mice, and plotting the resulting m values against the logarithm of the LD_50_ units on wire.

## Figures and Tables

**Table 1.** Both RML- and vCJD-associated PrP displayed double exponential decays when incubated with a fixed concentration of PK RML-infected brain contained the greater proportion of a highly resistant fraction at 82.7 % compared with 60 % in vCJD. In addition to being more abundant, the slowly degraded fraction associated with RML infection was more resistant to digestion than the equivalent fraction in vCJD brain, being degraded almost three times slower at 0.0004 s ^−1^ compared with 0.0011 s^−1^ for vCJD.

	**RML**	**vCJD**
Fast phase amplitude	21.8	46.4
Fast phase rate (s^−1^)	0.13	0.06
Slow phase amplitude	82.7	59.6
Slow phase rate (s^−1^)	0.0004	0.0011

**Table 2.** Titration comparisons of RML-infected brain homogenate by standard bioassay, implantation of wires and SSBA

**Dilution of RML brain**	**Treatment**	**RML prion titre by Tga20 mouse bioassay (i.c. LD_50_ units ml^−1^)**	**RML prion wire titre by i.c. implantation into Tg20 mice (i.c. LD_50_ wire units)**	**TCIU_w_ (estimated by SSBA) normalized to 1000 cells (mean±sem)**
10^−1^	None	10^8.3^	10^5.5^	–
10^−2^	None	10^7.3^	10^4.5^	–
10^−3^	None	10^6.3^	10^3.5^	–
10^−4^	None	10^5.3^	10^2.5^	>3.1
10^−5^	None	10^4.3^	10^1.5^	0.57±0.05
10^−6^*	None	10^3.3^	10^0.5^	0.28±0.02
10^−7^	None	10^2.3^	–	0.15±0.02
10^−8^†	None	10^1.3^	−	0.12±0.02
10^−9^	None	–	−	0.04±0.01
10^−10^	None	–	−	0.0029±0.002
10^−2^	Rely^+^On PI	–	<10^0.5^	<0.0029
10^−2^	HAMO 100 (0.8 % v/v)	–	−	0.2963
10^−2^	HAMO 100 (1.6 % v/v)	–	−	0.0725
10^−2^	Prionzyme	–	−	<0.0029
10^−2^	2 M NaOH	–	−	<0.0029
10^−2^	Autoclave at 134 °C	–	−	0.0325

*Limit of detection of RML prion infectivity in tga20 by wire i.c. inoculation. †Limit of detection of RML prion infectivity in tga20 by conventional i.c. inoculation of brain homogenate.

**Table 3.** Bioassay data from tga20 mice inoculated i.c. with 5 mm Steelex monofilament wires (USP4/0), exposed to a serial dilution of RML-infected brain homogenate Animals inoculated with wires treated with Rely^+^On PI (as described in Methods) survived to the end point of the experiment (>250 days post-inoculation). Subsequent Western blot and immunohistochemistry (IHC) analysis confirmed no signs of subclinical infection.

**Dilution of RML brain**	**Treatment of wires prior to inoculation**	Animals affected*†/total‡	**Mean incubation period (days±sem)**
10^−1^	None	20/20	76±1
10^−2^	None	18/18	86±2
10^−3^	None	18/18	85±2
10^−4^	None	18/18	113±9
10^−5^	None	28/35	114±7
10^−6^	None	4/18	157±24
10^−7^	None	0/19	>250
10^−8^	None	0/20	>250
PBS control	None	0/20	>250
10^−1^	Rely^+^On PI	0/19	>250

*All clinically affected animals were confirmed prion-infected by Western blot and IHC analysis for the presence of PrP^Sc^. †Clinically unaffected animals showed no signs of subclinical infection by Western blot or IHC analysis. ‡Variation in experimental group sizes due to intermittent losses for health reasons other than prion infection.

## References

[r1] Asante, E. A., Linehan, J. M., Desbruslais, M., Joiner, S., Gowland, I., Wood, A. L., Welch, J., Hill, A. F., Lloyd, S. E. & other authors (2002). BSE prions propagate as either variant CJD-like or sporadic CJD-like prion strains in transgenic mice expressing human prion protein. EMBO J 21, 6358–6366. 12456643 10.1093/emboj/cdf653 PMC136957

[r2] Asante, E. A., Gowland, I., Grimshaw, A., Linehan, J. M., Smidak, M., Houghton, R., Osiguwa, O., Tomlinson, A., Joiner, S. & other authors (2009). Absence of spontaneous disease and comparative prion susceptibility of transgenic mice expressing mutant human prion proteins. J Gen Virol 90, 546–558. 19218199 10.1099/vir.0.007930-0 PMC2885063

[r3] Bernoulli, C., Siegfried, J., Baumgartner, G., Regli, F., Rabinowicz, T., Gajdusek, D. C. & Gibbs, C. J., Jr (1977). Danger of accidental person-to-person transmission of Creutzfeldt-Jakob disease by surgery. . Lancet 1, 478–479. 10.1016/s0140-6736(77)91958-4 65575

[r4] Bramble, M. G. & Ironside, J. W. (2002). Creutzfeldt-Jakob disease: implications for gastroenterology. Gut 50, 888–890. 12010896 10.1136/gut.50.6.888 PMC1773231

[r5] Bruce, M. E., McConnell, I., Will, R. G. & Ironside, J. W. (2001). Detection of variant Creutzfeldt–Jakob disease infectivity in extraneural tissues. Lancet 358, 208–209. 11476840 10.1016/s0140-6736(01)05411-3

[r6] Bueler, H., Aguzzi, A., Sailer, A., Greiner, R. A., Autenried, P., Aguet, M. & Weissmann, C. (1993). Mice devoid of PrP are resistant to scrapie. Cell 73, 1339–1347. 8100741 10.1016/0092-8674(93)90360-3

[r7] Clewley, J. P., Kelly, C. M., Andrews, N., Vogliqi, K., Mallinson, G., Kaisar, M., Hilton, D. A., Ironside, J. W., Edwards, P. & other authors (2009). Prevalence of disease related prion protein in anonymous tonsil specimens in Britain: cross sectional opportunistic survey. BMJ 338, b1442. 10.1136/bmj.b1442 PMC2685439 19460798

[r8] Collinge, J. (2001). Prion diseases of humans and animals: their causes and molecular basis. Annu Rev Neurosci 24, 519–550. 11283320 10.1146/annurev.neuro.24.1.519

[r9] Collinge, J. & Clarke, A. R. (2007). A general model of prion strains and their pathogenicity. Science 318, 930–936. 17991853 10.1126/science.1138718

[r10] Collins, S., Law, M. G., Fletcher, A., Boyd, A., Kaldor, J. & Masters, C. L. (1999). Surgical treatment and risk of sporadic Creutzfeldt–Jakob disease: a case-control study. Lancet 353, 693–697. 10073510 10.1016/s0140-6736(98)08138-0

[r11] Cronier, S., Gros, N., Tattum, M. H., Jackson, G. S., Clarke, A. R., Collinge, J. & Wadsworth, J. D. (2008). Detection and characterization of proteinase K-sensitive disease-related prion protein with thermolysin. Biochem J 416, 297–305. 18684106 10.1042/BJ20081235 PMC2584334

[r12] Davanipour, Z., Alter, M., Sobel, E. & Callahan, M. (1985). Sheep consumption: a possible source of spongiform encephalopathy in humans. Neuroepidemiology 4, 240–249. 3915057 10.1159/000110235

[r13] de Pedro-Cuesta, J., Mahillo-Fernandez, I., Rabano, A., Calero, M., Cruz, M., Siden, A., Laursen, H., Falkenhorst, G. & Molbak, K. (2010). Nosocomial transmission of sporadic Creutzfeldt-Jakob disease: results from a risk-based assessment of surgical interventions. J Neurol Neurosurg Psychiatry (14 June; Epub ahead of print). 10.1136/jnnp.2009.188425 PMC3022351 20547628

[r14] Edgeworth, J. A., Jackson, G. S., Clarke, A. R., Weissmann, C. & Collinge, J. (2009). Highly sensitive, quantitative cell-based assay for prions adsorbed to solid surfaces. Proc Natl Acad Sci U S A 106, 3479–3483. 19204279 10.1073/pnas.0813342106 PMC2637901

[r15] Fichet, G., Comoy, E., Duval, C., Antloga, K., Dehen, C., Charbonnier, A., McDonnell, G., Brown, P., Lasmezas, C. I. & Deslys, J. P. (2004). Novel methods for disinfection of prion-contaminated medical devices. Lancet 364, 521–526. 15302195 10.1016/S0140-6736(04)16810-4

[r16] Fischer, M., Rulicke, T., Raeber, A., Sailer, A., Moser, M., Oesch, B., Brandner, S., Aguzzi, A. & Weissmann, C. (1996). Prion protein (PrP) with amino-proximal deletions restoring susceptibility of PrP knockout mice to scrapie. EMBO J 15, 1255–1264. 8635458 PMC450028

[r17] Flechsig, E., Hegyi, I., Enari, M., Schwarz, P., Collinge, J. & Weissmann, C. (2001). Transmission of scrapie by steel-surface-bound prions. Mol Med 7, 679–684. 11713367 PMC1949999

[r18] Frosh, A., Smith, L. C., Jackson, C. J., Linehan, J., Brandner, S., Wadsworth, J. D. & Collinge, J. (2004). Analysis of 2000 consecutive UK tonsillectomy specimens for disease-related prion protein. Lancet 364, 1260–1262. 15464187 10.1016/S0140-6736(04)17143-2

[r19] Gibbs, C. J., Jr, Asher, D. M., Kobrine, A., Amyx, H. L., Sulima, M. P. & Gajdusek, D. C. (1994). Transmission of Creutzfeldt–Jakob disease to a chimpanzee by electrodes contaminated during neurosurgery. J Neurol Neurosurg Psychiatry 57, 757–758. 8006664 10.1136/jnnp.57.6.757 PMC1072988

[r20] Giles, K., Glidden, D. V., Beckwith, R., Seoanes, R., Peretz, D., DeArmond, S. J. & Prusiner, S. B. (2008). Resistance of bovine spongiform encephalopathy (BSE) prions to inactivation. PLoS Pathog 4, e1000206. 19008948 10.1371/journal.ppat.1000206 PMC2576443

[r21] Glatzel, M., Abela, E., Maissen, M. & Aguzzi, A. (2003). Extraneural pathologic prion protein in sporadic Creutzfeldt–Jakob disease. N Engl J Med 349, 1812–1820. 14602879 10.1056/NEJMoa030351

[r22] Griffith, J. S. (1967). Self replication and scrapie. Nature 215, 1043–1044. 4964084 10.1038/2151043a0

[r23] Head, M. W., Ritchie, D., Smith, N., McLoughlin, V., Nailon, W., Samad, S., Masson, S., Bishop, M., McCardle, L. & Ironside, J. W. (2004). Peripheral tissue involvement in sporadic, iatrogenic, and variant Creutzfeldt–Jakob disease: an immunohistochemical, quantitative, and biochemical study. Am J Pathol 164, 143–153. 14695328 10.1016/S0002-9440(10)63105-7 PMC1602214

[r24] Hernandez-Palazon, J., Martinez-Lage, J. F., Tortosa, J. A. & Garcia-Cayuela, J. M. (1998). Anaesthetic management in patients suspected of, or at risk of, having Creutzfeldt–Jakob disease. Br J Anaesth 80, 516–518. 9640162 10.1093/bja/80.4.516

[r25] Hill, A. F., Desbruslais, M., Joiner, S., Sidle, K. C., Gowland, I., Collinge, J., Doey, L. J. & Lantos, P. (1997). The same prion strain causes vCJD and BSE. Nature 389, 448–450, 526. 9333232 10.1038/38925

[r26] Hill, A. F., Butterworth, R. J., Joiner, S., Jackson, G. S., Rossor, M. N., Thomas, D. J., Frosh, A., Tolley, N., Bell, J. E. & other authors (1999). Investigation of variant Creutzfeldt–Jakob disease and other human prion diseases with tonsil biopsy samples. Lancet 353, 183–189. 9923873 10.1016/s0140-6736(98)12075-5

[r27] Hilton, D. A., Ghani, A. C., Conyers, L., Edwards, P., McCardle, L., Ritchie, D., Penney, M., Hegazy, D. & Ironside, J. W. (2004). Prevalence of lymphoreticular prion protein accumulation in UK tissue samples. J Pathol 203, 733–739. 15221931 10.1002/path.1580

[r28] HPA Press Office (2009). vCJD abnormal prion protein found in a patient with haemophilia at post mortem.

[r29] Jackson, G. S., McKintosh, E., Flechsig, E., Prodromidou, K., Hirsch, P., Linehan, J., Brandner, S., Clarke, A. R., Weissmann, C. & Collinge, J. (2005). An enzyme-detergent method for effective prion decontamination of surgical steel. J Gen Virol 86, 869–878. 15722550 10.1099/vir.0.80484-0

[r30] Joiner, S., Linehan, J., Brandner, S., Wadsworth, J. D. & Collinge, J. (2002). Irregular presence of abnormal prion protein in appendix in variant Creutzfeldt–Jakob disease. J Neurol Neurosurg Psychiatry 73, 597–598. 12397162 10.1136/jnnp.73.5.597 PMC1738116

[r31] Joiner, S., Linehan, J., Brandner, S., Wadsworth, J. D. & Collinge, J. (2005). High Levels of disease related prion protein in the ileum in variant Creutzfeldt–Jakob disease. Gut 54, 1506–1508. 10.1136/gut.2005.072447 PMC1774705 16162963

[r32] Klohn, P. C., Stoltze, L., Flechsig, E., Enari, M. & Weissmann, C. (2003). A quantitative, highly sensitive cell-based infectivity assay for mouse scrapie prions. Proc Natl Acad Sci U S A 100, 11666–11671. 14504404 10.1073/pnas.1834432100 PMC208815

[r33] Lemmer, K., Mielke, M., Kratzel, C., Joncic, M., Oezel, M., Pauli, G. & Beekes, M. (2008). Decontamination of surgical instruments from prions. II. In vivo findings with a model system for testing the removal of scrapie infectivity from steel surfaces. J Gen Virol 89, 348–358. 18089760 10.1099/vir.0.83396-0

[r34] Llewelyn, C. A., Hewitt, P. E., Knight, R. S., Amar, K., Cousens, S., Mackenzie, J. & Will, R. G. (2004). Possible transmission of variant Creutzfeldt–Jakob disease by blood transfusion. Lancet 363, 417–421. 14962520 10.1016/S0140-6736(04)15486-X

[r35] Mahillo-Fernandez, I., Pedro-Cuesta, J., Bleda, M. J., Cruz, M., Molbak, K., Laursen, H., Falkenhorst, G., Martinez-Martin, P. & Siden, A. (2008). Surgery and risk of sporadic Creutzfeldt–Jakob disease in Denmark and Sweden: registry-based case-control studies. Neuroepidemiology 31, 229–240. 18843192 10.1159/000163097 PMC2790765

[r36] Minatogawa, T. & Kumoi, T. (1999). Problems in utility and safety of otological allografts. Transplant Proc 31, 2036–2037. 10455965 10.1016/s0041-1345(99)00258-4

[r37] Murdoch, H., Taylor, D., Dickinson, J., Walker, J. T., Perrett, D., Raven, N. D. & Sutton, J. M. (2006). Surface decontamination of surgical instruments: an ongoing dilemma. J Hosp Infect 63, 432–438. 16759745 10.1016/j.jhin.2006.02.015

[r38] Notari, S., Moleres, F. J., Hunter, S. B., Belay, E. D., Schonberger, L. B., Cali, I., Parchi, P., Shieh, W. J., Brown, P. & other authors (2010). Multiorgan detection and characterization of protease-resistant prion protein in a case of variant CJD examined in the United States. PLoS ONE 5, e8765. 20098730 10.1371/journal.pone.0008765 PMC2808239

[r39] Peden, A. H., Head, M. W., Ritchie, D. L., Bell, J. E. & Ironside, J. W. (2004). Preclinical vCJD after blood transfusion in a PRNP codon 129 heterozygous patient. Lancet 364, 527–529. 15302196 10.1016/S0140-6736(04)16811-6

[r40] Peden, A., McCardle, L., Head, M. W., Love, S., Ward, H. J., Cousens, S. N., Keeling, D. M., Millar, C. M., Hill, F. G. & Ironside, J. W. (2010). Variant CJD infection in the spleen of a neurologically asymptomatic UK adult patient with haemophilia. Haemophilia 16, 296–304. 20070383 10.1111/j.1365-2516.2009.02181.x

[r41] Peretz, D., Supattapone, S., Giles, K., Vergara, J., Freyman, Y., Lessard, P., Safar, J. G., Glidden, D. V., McCulloch, C. & other authors (2006). Inactivation of prions by acidic sodium dodecyl sulfate. J Virol 80, 322–331. 16352557 10.1128/JVI.80.1.322-331.2006 PMC1317507

[r42] Prusiner, S. B. (1982). Novel proteinaceous infectious particles cause scrapie. Science 216, 136–144. 6801762 10.1126/science.6801762

[r43] Prusiner, S. B., Groth, D. F., McKinley, M. P., Cochran, S. P., Bowman, K. & Kasper, K. C. (1981). Thiocyanate and hydroxyl ions inactivate the scrapie agent. Proc Natl Acad Sci U S A 78, 4606–4610. 6794034 10.1073/pnas.78.7.4606 PMC319842

[r44] Rutala, W. A. & Weber, D. J. (2001). Creutzfeldt–Jakob disease: recommendations for disinfection and sterilization. Clin Infect Dis 32, 1348–1356. 11303271 10.1086/319997

[r45] SEAC (2006). Position statement on methods to evaluate decontamination technologies for surgical instruments. http://www.seac.gov.uk/statements/statement310806.htm.

[r46] Smith, A., Dickson, M., Aitken, J. & Bagg, J. (2002). Contaminated dental instruments. J Hosp Infect 51, 233–235. 12144804 10.1053/jhin.2002.1213

[r47] Somerville, R. A., Oberthur, R. C., Havekost, U., MacDonald, F., Taylor, D. M. & Dickinson, A. G. (2002). Characterization of thermodynamic diversity between transmissible spongiform encephalopathy agent strains and its theoretical implications. J Biol Chem 277, 11084–11089. 11792707 10.1074/jbc.M111766200

[r48] Taylor, D. M. (1991). Inactivation of the unconventional agents of scrapie, bovine spongiform encephalopathy and Creutzfeldt–Jakob disease. J Hosp Infect 18, 141–146 (Suppl. A). 1679777 10.1016/0195-6701(91)90016-2

[r49] Taylor, D. M., Fernie, K., McConnell, I. & Steele, P. J. (1998). Observations on thermostable subpopulations of the unconventional agents that cause transmissible degenerative encephalopathies. Vet Microbiol 64, 33–38. 9874101 10.1016/s0378-1135(98)00257-0

[r50] Taylor, D. M., Fernie, K., McConnell, I. & Steele, P. J. (1999). Survival of scrapie agent after exposure to sodium dodecyl sulphate and heat. Vet Microbiol 67, 13–16. 10392773 10.1016/s0378-1135(99)00026-7

[r51] Wadsworth, J. D., Joiner, S., Hill, A. F., Campbell, T. A., Desbruslais, M., Luthert, P. J. & Collinge, J. (2001). Tissue distribution of protease resistant prion protein in variant CJD using a highly sensitive immuno-blotting assay. Lancet 358, 171–180. 11476832 10.1016/s0140-6736(01)05403-4

[r52] Wadsworth, J. D., Joiner, S., Fox, K., Linehan, J., Desbruslais, M., Brandner, S., Asante, E. A. & Collinge, J. (2007). Prion infectivity in variant Creutzfeldt–Jakob disease rectum. Gut 56, 90–94. 16763054 10.1136/gut.2006.091637 PMC1856674

[r53] Walker, J. T., Dickinson, J., Sutton, J. M., Raven, N. D. & Marsh, P. D. (2007). Cleanability of dental instruments – implications of residual protein and risks from Creutzfeldt–Jakob disease. Br Dent J 203, 395–401. 17934424 10.1038/bdj.2007.893

[r54] Ward, H. J., Everington, D., Croes, E. A., Alperovitch, A., Delasnerie-Laupretre, N., Zerr, I., Poser, S. & van Duijn, C. M. (2002). Sporadic Creutzfeldt–Jakob disease and surgery: a case-control study using community controls. Neurology 59, 543–548. 12196646 10.1212/wnl.59.4.543

[r55] WHO (1999). WHO Infection Control Guidelines for Transmissible Spongiform Encephalopathies. Geneva. : WHO.

[r56] Wroe, S. J., Pal, S., Siddique, D., Hyare, H., Macfarlane, R., Joiner, S., Linehan, J., Brandner, S., Wadsworth, J. D. & other authors (2006). Clinical presentation and pre-mortem diagnosis of variant Creutzfeldt–Jakob disease associated with blood transfusion: a case report. Lancet 368, 2061–2067. 17161728 10.1016/S0140-6736(06)69835-8

[r57] Yan, Z. X., Stitz, L., Heeg, P., Pfaff, E. & Roth, K. (2004). Infectivity of prion protein bound to stainless steel wires: a model for testing decontamination procedures for transmissible spongiform encephalopathies. Infect Control Hosp Epidemiol 25, 280–283. 15108723 10.1086/502392

[r58] Zobeley, E., Flechsig, E., Cozzio, A., Masato, E. & Weissmann, C. (1999). Infectivity of scrapie prions bound to a stainless steel surface. Mol Med 5, 240–243. 10448646 PMC2230327

